# Laparoscopic removal of gastric balloon after failure of endoscopic retrieval

**DOI:** 10.1016/j.ijscr.2019.01.043

**Published:** 2019-02-10

**Authors:** Mohammed Sharroufna, Ali Hassan, Marwah Alabdrabalmeer, Saeed Alshomimi

**Affiliations:** Department of Surgery, King Fahd Hospital of The University, Imam Abdulrahman Bin Faisal University, Dammam, Saudi Arabia

**Keywords:** Gastric, Balloon, Retrieval, Laparoscopic

## Abstract

**Introduction:**

Different therapeutic measures have been developed in the treatment of obesity. Gastric balloon is a minimally-invasive modality in obesity treatment, but it is not without a risk of complications.

**Presentation of case:**

We present a 44-year-old morbidly obese lady who underwent gastric balloon insertion and refused to remove it at the recommended time. Unfortunately, after 18 months from insertion of the balloon, she was brought to the Emergency Department with symptoms of gastric outlet obstructions. Endoscopic retrieval of the balloon has failed. Hence, surgical intervention was planned and the balloon was successfully removed laparoscopically.

**Discussion:**

Gastric balloons are designed to remain in the stomach for 6 months. Delayed extraction of the balloon associated with increase in the rate of complications. In our case, it was difficult to retrieve the balloon endoscopically due to thickened balloon wall, which was then removed laparoscopically safely without any complications.

**Conclusion:**

Laparoscopic extraction of the gastric balloon is a safe and feasible option in the management of difficult endoscopic retrieval.

## Highlights

• Delayed extraction of gastric balloons increase rate of complications.
• Failure of endoscopic retrieval is a rare complication.
• Laparoscopic retrieval of gastric balloon is an option in the management.

## 1 Introduction

Obesity is a major healthcare problem worldwide. It is associated with a significant morbidity and mortality. Different therapeutic measures have been developed for the management of obesity, including behavioral modifications, pharmacologic therapy, bariatric surgeries and other minimally-invasive procedures such as gastric balloons. Gastric balloons are non-surgical procedures but they are not without risk of complications (e.g. perforation, obstruction and balloon rupture). We present a case of a complicated gastric balloon which required surgical intervention after failure of endoscopic retrieval. This work has been done in line with the SCARE criteria [1].

## 2 Presentation of case

We report a case of a 44-year-old female patient with morbid obesity (height 152 cm, weight 117 kg, BMI 50.6 kg/m^2^), hypertension and rheumatoid arthritis who attempted several cycles of lifestyle modifications for weight loss but the result was suboptimal. As a result, she was proposed for gastric balloon insertion. The patient was aiming to lose more weight, so she refused to remove the balloon 6 months after its insertion, going against the medical advice.

Unfortunately, after 18 months from gastric balloon insertion, our patient was brought to the emergency department with 3 days history of nausea and repeated episodes of vomiting. The physical examination was unremarkable. On admission, it showed that she had lost 32 kg of her weight, reaching a BMI of 36.8 kg/m^2^. Endoscopic retrieval of the balloon was decided and the patient kept on nil per oral and on intravenous fluids.

With the patient under conscious sedation (monitored anesthesia care), the endoscopist had punctured and deflated the balloon but could not extract it, as it disengaged on reaching the pharynx. Despite the use of muscle relaxants, several attempts have failed to extract the balloon due to the short neck of patient, thickened deformed balloon and tight pharyngeal muscle. Hence, the surgery team was consulted regarding the possibility of surgical intervention.

**Fig. 1:**
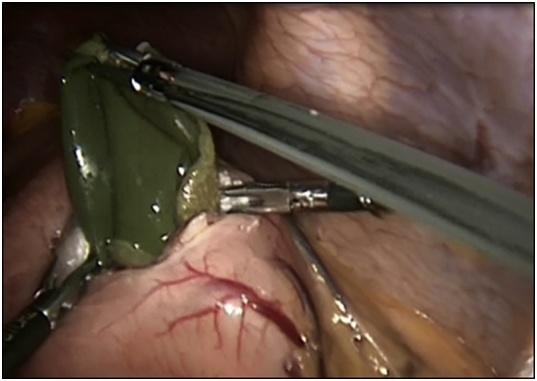
Laparoscopic view during the retrieval of the gastric balloon.

**Fig. 2:**
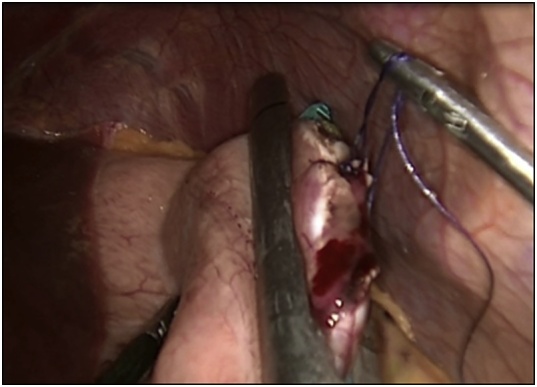
Laparoscopic view during closure of gastrotomy after gastric balloon removal.

**Fig. 3:**
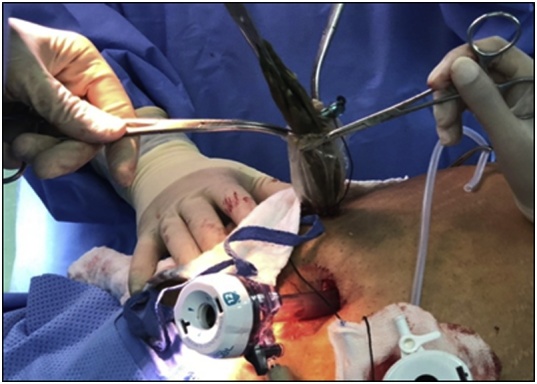
Removal of gastric balloon using Endobag? through the 12-mm port.

**Fig. 4:**
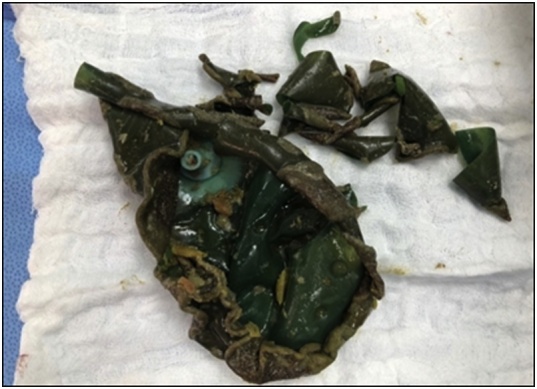
The balloon after successful extraction with its thickened deformed wall.

The patient was prepared for emergency laparoscopic gastrotomy and balloon removal. Four ports were inserted for carrying out the procedure. Stomach was deflated by nasogastric tube, and gastrotomy opening was done laterally in the greater curvature side using Harmonic device away from a possible future bariatric surgery plane. Balloon was extracted using Endo Clinch™ (Fig. 1) and the gastrotomy closed using Ethicon Green Stapler (Fig. 2). Methylene blue was flushed into the stomach through nasogastric tube and no leak was seen. Balloon was removed using Endobag™ through 12-mm port (Fig. 3, Fig. 4). Postoperative recovery was uneventful. She had Gastrograffin study on the first post-operative day and it showed no evidence of leak. Two weeks later, the patient was reassessed in the outpatient clinic where she was completely asymptomatic.

## 3 Discussion

Since early 1980s, gastric balloons have been used as a temporary non-surgical measure for weight reduction in patients who are not candidates for bariatric surgery or as bridging intervention prior to bariatric surgery in morbidly obese patients [2]. The gastric balloons induce weight loss by working as a restrictive measure by inducing early satiety [3] and it may delay the gastric emptying [4].

The earliest balloons introduced to the market were air-filled with sharp edges and low maximum fill volumes found to be associated with high rates of complications and failure in achieving adequate weight loss [2]. In early 1990s, a new balloon was introduced to the market, the BioEnterics Intragastric Balloon (BIB^®^; Santa Barbara, CA, USA), based on the recommendations of a scientific conference held in Tapron Springs, Florida, 1987 which defined the fundamental features of the ideal gastric balloon [2,5]. The BIB^®^ is made of high quality silicone, that is filled with 500–700 mL of saline plus 10 mL of methylene blue (to identify leakage or rupture) once endoscopically placed in the stomach, forming a smooth surface sphere. It also has a radio-opaque valve with self-sealing mechanism that allow radiological visualization of the balloon [6].

With the use of fluid-filled gastric balloons, number of adverse events have been reported. Majority of the patients will develop mild gastrointestinal symptoms including nausea and vomiting, abdominal pain and reflux. Less common serious adverse effects may also develop in some patients such as balloon rupture, balloon migration with possible intestinal obstruction, bleeding ulcers, gastric outlet obstruction and gastric perforation [7,8].

Commonly used gastric balloons are designed to remain in the stomach for a maximum duration of 6 months. Although a multi-centric Italian study reported that BIB^®^ treatment up to 14 months was found to achieve greater weight loss than the BIB^®^ in situ for 6 months without complications [9], early gastric balloon extraction is vital to minimize complications [10]. Balloons retained for more than 6 months found to be associated with higher rates of balloon rupture, displacement and occasional intestinal obstruction [10].

Regarding the endoscopic removal of gastric balloons, it was found that double-channel gastroscope and rat-toothed forceps plus symmetrical shark polypectomy snare allows safe removal of the balloon with a short retrieval time [11]. However, in our situation, double-channel endoscope was not available and the standard gastroscope was used. An alternative approach for difficult retrieval was proposed by Neto et al with spraying vegetable oil over the balloon and throughout the esophagus to facilitate the extraction of balloon with decreased risk of esophageal injury [12].

In our case, delaying of balloon retrieval to 18 months may lead to thickening of the balloon wall and difficulty in endoscopic extraction which may require surgical intervention.

## Conflict of interest

Nothing to disclose.

## Sources of funding

No source of funding.

## Ethical approval

No ethical approval needed; case report would be published without any identification data.

## Consent

Written consent was taken from the patient herself.

## Author contribution

Mohammed Sharroufna: writing the paper.

Ali Hassan: writing the paper.

Marwah Nasser E Albdrabalameer: writing the paper.

Saeed Alshomimi: supervisor; editing the paper; treating physician of the patient.

## Registration of research studies

Not Applicable (Case Report; not an interventional study) and not needed by our institute.

## Guarantor

Dr. Saeed AlShomimi.

## Provenance and peer review

Not commissioned, externally peer-reviewed.
